# Engineering, and production of functionally active human Furin in *N*. *benthamiana* plant: *In vivo* post-translational processing of target proteins by Furin in plants

**DOI:** 10.1371/journal.pone.0213438

**Published:** 2019-03-12

**Authors:** Tarlan Mamedov, Ilaha Musayeva, Rabia Acsora, Nilufer Gun, Burcu Gulec, Gulshan Mammadova, Kader Cicek, Gulnara Hasanova

**Affiliations:** 1 Akdeniz University, Department of Agricultural Biotechnology, Antalya, Turkey; 2 Azerbaijan National Academy of Science, Department of Biology and Medical Science, Baku, Azerbaijan; National Botanical Research Institute CSIR, INDIA

## Abstract

A plant expression platform with eukaryotic post-translational modification (PTM) machinery has many advantages compared to other protein expression systems. This promising technology is useful for the production of a variety of recombinant proteins including, therapeutic proteins, vaccine antigens, native additives, and industrial enzymes. However, plants lack some of the important PTMs, including furin processing, which limits this system for the production of certain mammalian complex proteins of therapeutic value. Furin is a ubiquitous proprotein convertase that is involved in the processing (activation) of a wide variety of precursor proteins, including blood coagulation factors, cell surface receptors, hormones and growth factors, viral envelope glycoproteins, etc. and plays a critical regulatory role in a wide variety of cellular events. In this study, we engineered the human furin gene for expression in plants and demonstrated the production of a functional active recombinant truncated human furin in *N*. *benthamiana* plant. We demonstrate that plant produced human furin is highly active both *in vivo* and *in vitro* and specifically cleaved the tested target proteins, Factor IX (FIX) and Protective Antigen (PA83). We also demonstrate that both, enzymatic deglycosylation and proteolytic processing of target proteins can be achieved *in vivo* by co-expression of deglycosylating and furin cleavage enzymes in a single cell to produce deglycosylated and furin processed target proteins. It is highly expected that this strategy will have many potential applications in pharmaceutical industry and can be used to produce safe and affordable therapeutic proteins, antibodies, and vaccines using a plant expression system.

## Introduction

In recent years, numerous studies have demonstrated the plant transient expression systems as a promising expression platform with high expression capacity, which provide safe, cost-effective production of a variety of biologically active proteins in a relatively short period of time [1–5]. Since plants possess eukaryotic PTM machinery, this technology is especially useful for the production of mammalian complex proteins, where PTMs play a critical role in the proper folding and functional activity [6]. Despite the significant similarities between mammalian and plant cell PTM machinery, plants lack the ability to make a number of important PTMs present in mammalian cells [6]. Furin is a mammalian subtilisin/kex2p like endoprotease, belonging to the proprotein convertase family. It is responsible for the post-translational cleavage of a number of precursor proteins. Although the existence of a kex2p like pathway was reported earlier in *Nicotiana tabacum* plant [7], no furin cleavage activity was observed in plants [8]. The human furin protein is a 794 amino acid long, membrane-associated protease. It possesses a signal peptide, propeptide, and subtilisin-like catalytic domain characterized by a catalytic triad of three amino acids: aspartate, histidine and serine. Furin also contains a cysteine rich domain, homo B domain, which is essential for catalytic activity. It is anchored in the plasma membrane by a transmembrane domain and the cytoplasmic domain, which regulates the localization of furin in the cellular trans-Golgi network [9]. The luminal and extracellular domains of human furin share a homology with other members of the proprotein convertase (PC) family. Notably, the highest sequence similarity is found in the subtilisin-like catalytic domain, where aspartate, histidine and serine residues form a strictly conserved catalytic triad [10]. The catalytic domain of furin is 54–70% identical to other PCs [10]. Furin cleavage of propeptides is essential for maturation of the precursor proteins. For example, furin processing is essential for the gamma carboxylation of glutamic acid residues [11], disulfide bridge formation [12, 13], regulating the synthesis of multiple mature peptides [14, 15], and directing intracellular targeting [16]. Furin is involved in the cleavage of serum proteins including blood clotting factors, cell surface receptors, hormones, growth factors and their receptors [9, 17] mainly at Arg-*X*-Lys/Arg-Arg (R*X*K/RR) consensus sequence [18,19]. A number of studies have reported that a mutation found at the furin cleavage site of certain precursor proteins is associated with the onset of the various types of diseases [9]. For example, the envelope proteins of influenza virus [20], HIV [21], dengue fever [22] and several filoviruses including ebola and marburg virus [23] must be cleaved by furin-like proteases to become functionally active. Furin also functions in the cleavage of papillomaviruses [24], anthrax toxin [25] and pseudomonad exotoxin [26] during entry into the host cells. It should be noted that, recombinant blood clotting factors VII, VIII, IX, and protein C, which are currently used in the treatment of a number of diseases are mainly prepared from donated human blood and therefore, have a viral contamination risk and also very expensive [27–29]. Defects in factor IX (FIX) synthesis result in hemophilia B (Christmas disease), an X-linked disorder. Different expression systems have been used to produce recombinant FIX [30], however, all attempts were impeded by limitations in PTM, safety, and high costs. A plant transient expression system could be an alternative expression system for the production of safe and affordable blood clotting factors, such as FIX to be used as a hemophilia B treatment. As previously stated, plants lack a number of important mammalian PTMs, such as gamma carboxylation, furin processing, sialylation, mannose-6-phosphate modification, sulfation, and etc. [6]. Therefore, engineering of plants that can produce recombinant proteins in their native forms would be very important for their functionality. FIX is expressed as a precursor polypeptide that requires posttranslational processing. In order to produce functionally active FIX in plants, FIX must be cleaved *in vivo* by PACE (Paired basic Amino acid Cleaving Enzyme)/furin processing enzyme. Given that furin cleavage is involved in many different cellular events, production of functionally active furin is necessary for the production of a variety of pharmaceutically valuable precursor proteins. Since human furin is a transmembrane protein, it therefore, would be challenging to produce a highly soluble and fully functional active human furin in plants. At this point, although co-expression of full length human furin and latent transforming growth factor-b1 in *N*. *benthamiana* plants was recently reported [8], there was no direct confirmation of furin expression; for example, western blot analysis using a specific antibody of plant produced human furin was not reported in the study [8]. Additionally, there were no reports regarding the isolation of furin from plants or the *in vitro* assessment of plant produced recombinant furin. In this study, we engineered a human furin gene for production in plants. For the first time we have shown that this truncated version of recombinant human furin is highly expressed, soluble and functionally active in *N*. *benthamiana* plants. We also described a method for producing furin processed proteins in plant cells by co-expressing human furin with target proteins of interest *in vivo*. We transiently co-expressed a hemophilia B therapeutic candidate, FIX, and an anthrax vaccine candidate, protective antigen (PA) of *B*. *anthracis* along side human furin in *N*. *benthamiana*. Our results showed that human furin cleaved all target proteins. We also demonstrated that both the enzymatic deglycosylation and proteolytic processing of target proteins were achieved *in vivo* by introducing and co-expressing deglycosylating and furin cleavage enzymes in the same plant cells.

## Materials and methods

### Cloning, expression and production of recombinant human furin and FIX in *N*. *benthamiana* plants

Human furin and FIX genes were engineered for expression in *N*. *benthamiana* plants and synthesized at Biomatik Corporation. Full length furin (amino acids 26–794) was transiently expressed in *N*. *benthamiana* plants. The signal peptide (amino acids 1–25) was removed from the furin sequence (GenBank Accession No. NP_002560), and *N*. *tabacum* PR-1a signal peptide (MGFVLFSQLPSFLLVSTLLLFLVISHSCRA) was added to the N-terminus. The KDEL sequence (the ER retention signal) and the His6 tag (the affinity purification tag) were added to the C-terminus. A truncated form of furin (amino acids 26–595) was expressed with a PR-1a signal peptide at N-terminus and His6-KDEL at C-terminus. The resulting sequences were inserted into the pEAQ vector [31] using AgeI/XhoI sites resulting in pEAQ-Furin (full length)-His6-KDEL and pEAQ-Furin (truncated)-His6-KDEL constructs. To transiently express the FIX (amino acids 29–461) in *N*. *benthamiana* plants, the signal peptide (amino acids 1–28) was removed from the FIX sequence (GenBank Accession No. NP_000124) and replaced with a *N*. *tabacum* PR-1a signal peptide (MGFVLFSQLPSFLLVSTLLLFLVISHSCRA). Like the furin constructs, a KDEL sequence and the His6 tag were added to the C-terminus. The human FIX gene was also synthesized at Biomatik Corporation with a FLAG tag positioned at the N-terminal, between PR-1a signal peptide and propeptide sequence. These sequences were inserted into the pEAQ vector as previously described using the same restrictions sites resulting in pEAQ-FIX-His6-KDEL and pEAQ-FLAG-FIX-KDEL constructs. All of the expression constructs described above were then transformed into the *Agrobacterium tumefaciens* strain AGL1 using electroporation. Agrobacterium growth, plant growth, plant infiltration, plant leaf tissue harvesting, plant extraction, homogenization and further analysis were performed as described previously [4]. Leaf tissue was harvested at 4–7 dpi (day post infiltration) and homogenized in three volumes of extraction buffer (20 mM sodium phosphate, 150 mM sodium chloride, pH 7.4). After centrifugation of the crude extract at 13 000 g for 20 min samples were run on SDS-PAGE, and transferred to a Polyvinylidene fluoride (PVDF) membranes (Millipore, Billerica, MA) for western blot analysis. The His tagged proteins were probed with a purified mouse anti-His tag primary antibody (Cat. no. 652502, BioLegend) and anti-mouse horseradish peroxidase (HRP)-conjugated IgG secondary antibody (Cat. No. ab98790, Abcam). The FLAG tagged recombinant FIX protein was detected using anti-FLAG mAb (Cat. No.637301, BioLegend), followed by HRP goat anti-rat IgG antibody (Cat. No. 405405, BioLegend). *N*. *benthamiana* plants infiltrated with an empty pEAQ vector was used as a negative control. The pEAQ binary expression vector was kindly provided by Dr. George P. Lomonossoff (John Innes Centre, Biological Chemistry Department).

### Cloning and expression of Endo H, PNGase F and PA83 in *N*. *benthamiana*

Endo H (Endo-ß-N-acetylglucosaminidase), PNGase F (Peptide N-Glycosidase F) and *B*. *anthracis* PA83 genes were engineered for expression in plants, cloned and expressed as described recently [4]. Endo H or PNGase F sequences were inserted into the pBI121 expression vectors resulting in pBI-Endo H-FLAG-KDEL or pBI-PNGase F-FLAG-KDEL constructs. The PA83 gene sequence was inserted into the pEAQ or pBI vectors resulting in pEAQ-PA83-His6-KDEL or pBI-PA83-His6-KDEL constructs. Agrobacterium growth, plant growth, plant infiltration, plant leaf tissue harvesting, extraction, homogenization and further analysis were performed as described previously [4].

### Co-expression of human furin with FIX and PA83 *of B*. *anthracis* and evaluation of its cleavage activity *in vivo*

To co-express a human furin with FIX, pEAQ-Furin/pEAQ-FIX (His6 and FLAG tagged) constructs were used for agrobacterium mediated infiltration of *N*. *benthamiana* plant leaves. Similarly, furin was co-expressed with *B*. *anthracis* PA83, using pEAQ-Furin/pBI-PA83 constructs in the same manner. These constructs were transformed into *A*. *tumefaciens* AGL1, which was used to infiltrate into 6–7 week old *N*. *benthamiana* plants. This was conducted in the absence of an *A*. *tumefaciens* AGL1 strain harboring a silencing suppressor, since pEAQ vector contained both genes of interest and the suppressor of silencing in a single plasmid. In order to achieve a complete furin processing of FIX and *B*. *anthracis* PA83 proteins *in vivo*, plant infiltration was optimized using different ratios of *A*. *tumefaciens* strain AGL1, expressing furin or target proteins. Leaf samples were taken at 4–7 dpi and homogenized in three volumes of extraction buffer (20 mM sodium phosphate, 150 mM sodium chloride, pH 7.4) using a mortar and pestle. Samples were run on 10% SDS-PAGE, followed by western blotting as described above. Since both furin and PA83 are His6-tagged proteins, they were detected using a purified mouse anti-His tag antibody (BioLegend, Cat. no. 652502). To confirm the site-specific cleavage of FIX by furin, the anti-FLAG antibody (BioLegend, Cat. No. 637301) and anti-FIX antibody (Cat. no. F9-1020A, CoaChrom Diagnostica, Austria) were also used.

### Co-expression of human Furin with PA83 of *B*. *anthracis* and deglycosylation enzymes Endo H or PNGase F in *N*. *benthamiana*

To co-express the human furin, PA83 and deglycosylation enzymes, Endo H or PNGase F, pEAQ-Furin/pEAQ-PA83-His6-KDEL/pBI-Endo H or pEAQ-Furin/pEAQ-PA83-His6-KDEL/pBI-PNGase F constructs were used in the agrobacterium mediated infiltration of *N*. *benthamiana* leaves. Agrobacterium growth, plant growth, plant infiltration, plant leaf tissue harvesting, extraction and homogenization and western blot analysis were performed as described above. To test the effect of pH on oligomer formation, the protein extraction was performed as follows: *N*. *benthamiana* plants, which were infiltrated with i) PA83 and Endo H or ii) PA83, Endo H and furin constructs, were homogenized in PBS (pH 7.4) or in 20 mM MES-Tris buffer, pH 5.0, containing 145 mM NaCI. Boiled and un-boiled samples were then analyzed by western blotting.

### Co-expression of human Furin with deglycosylation enzymes Endo H or PNGase F

To co-express a human furin with Endo H or PNGase F, pEAQ-Furin/pBI-Endo H or pEAQ-Furin/pBI-PNGase F constructs were used for plant infiltration. Leaf tissue was harvested at 5 dpi and homogenized in three volumes of extraction buffer (20 mM sodium phosphate, 150 mM sodium chloride, pH 7.4) and samples were run on SDS-PAGE and transferred to PVDF membranes (Millipore, Billerica, MA) for western blot analysis as previously described. Western blot analysis was performed as described above using a purified mouse anti-His tag antibody (Cat. no. 652502, BioLegend) as a primary antibody and an anti-mouse horseradish peroxidase (HRP)- conjugated IgG as a secondary antibody (Cat. No. ab98790, Abcam).

### Purification of plant produced furin by Ni-NTA column

Plant produced furin was partially purified as described previously [4]. Briefly, 25 grams of frozen plant leaves, infiltrated with the pEAQ-Furin (truncated)-His6-KDEL construct, were ground using mortar and pestle in a phosphate extraction buffer (20 mM sodium phosphate, 300 mM sodium chloride, 10 mM imidazole, pH 7.4). The plant cell extract was filtered through Miracloth and centrifuged at 20,000 g for 25 minutes and then filtered through a 0.45 μm syringe filter (Millipore). The clarified total protein extract was then purified using HisPur Ni-NTA resin (Cat. No. 88221, Thermo Fisher Scientific) by following a previously used protocol [4]. Endo H or PNGase F deglycosylated and non-deglycosylated furin proteins were purified using the same procedure described above. These partially purified furin proteins were concentrated and stored at -80°C until used for experimentation.

### In vitro cleavage of PA83 and APRIL proteins with plant produced or commercial furin

To assess the *in vitro* cleavage activity of plant produced furin, a plant produced deglycosylated PA83 (dPA83) and APRIL (Cat no. SRP3189-20UG, Sigma) proteins were used as furin substrates. dPA83 was produced in *N*. *benthamiana* plants by co-expressing PA83 with Endo H, as described previously [4]. A total of 5 μg plant produced, Ni-NTA column purified dPA83 was incubated with the various concentrations of plant produced human furin (0, 1, 5, 25, 50, 100 ng) in 20 mM HEPES, 0.1% Triton X-100, 0.2 mM CaCl_2_, pH 7.5, at 25°C for 2 hours. To compare the *in vitro* activity of plant produced furin with commercial human furin, 5 μg plant produced, Ni-NTA column purified dPA83 was incubated with various concentrations (0, 1, 5, 25, 50, 100 ng) of commercial human furin (New England Biolabs, NEB), in 20 mM HEPES, 0.1% Triton X-100, 0.2 mM CaCl_2_, pH 7.5, at 25°C for 2 hours. To test the APRIL protein cleavage by plant produced furin, 5 μg APRIL protein was incubated with 25 ng plant produced or commercial furin (NEB, Cat. no P8077S) in 20 mM HEPES, 0.1% Triton X-100, 0.2 mM CaCl_2_, pH 7.5, at 25°C for 2 hours. dPA83 samples that were incubated with plant produced furin were run on SDS-PAGE followed by western blot analysis. dPA83 cleavage by furin (with plant produced or commercial furin) was analyzed by SDS-PAGE and western blotting using 4.5 μg or 100 ng of dPA83 protein from each sample, respectively. 2.5 μg of the APRIL protein, cleaved by plant produced or commercial recombinant furin was analyzed on SDS-PAGE.

For the assessment of *in vitro* cleavage activity of plant produced deglycosylated furin proteins, 5.0 μg dPA83 protein was incubated with the increasing concentrations (1, 5, 25, 50, 100 ng) of purified, recombinant Endo H or PNGase F deglycosylated human furin in 20 mM HEPES, 0.1% Triton X-100, 0.2 mM CaCl_2_, pH 7.5, at 25°C for 2 hours. The effect of *in vitro* deglycosylation on commercial furin enzyme activity, 5.0 μg of recombinant dPA83 with 50 ng of Endo H or 50 ng of PNGase F deglycosylated human furin as described above. Equal amount of the dPA83 protein (2.5 μg) from each sample was analyzed by SDS-PAGE.

### SDS-PAGE and western blot analysis

SDS-PAGE analysis of furin cleaved proteins was performed using 10–12% acrylamide gels as described previously [4]. SDS-PAGE was performed under reducing condition by preparing samples in 5X Laemmli Buffer (100 mM Tris, 2% SDS, 20% glycerol, 4% β-mercaptoethanol, pH 6.8). For western blot analysis, protein samples were run on 10–12% acrylamide gels, followed by transfer to a PVDF membrane (Millipore, Billerica, MA). The membranes were blocked using a 0.5% I-block solution (Applied Biosystems, Carlsbad, CA). His6 tagged furin, FIX and PA83 proteins were detected as described above. The membranes were washed with a Phosphate-buffered saline, containing 0.1% Tween- 20 (PBS-T). After washing, membranes were incubated with an anti-mouse horseradish peroxidase (HRP)- conjugated secondary antibody (Cat. No. ab98790, Abcam) or anti-rabbit HRP-conjugated secondary antibody (Cat. No. ab97051, Abcam).The images of protein bands were acquired using the GeneSnap software on a GeneGenome using a chemiluminescent substrate (SuperSignal West Pico, Thermo Fisher Scientific, Grand Island, NY) and quantified using the Gene Tools software (Syngene Bioimaging, UK).

## Results

### Engineering and production of recombinant human furin in *N*. *benthamiana* plants and purification of His tagged recombinant furin using the Ni-NTA column chromatography

**Fig 1:**
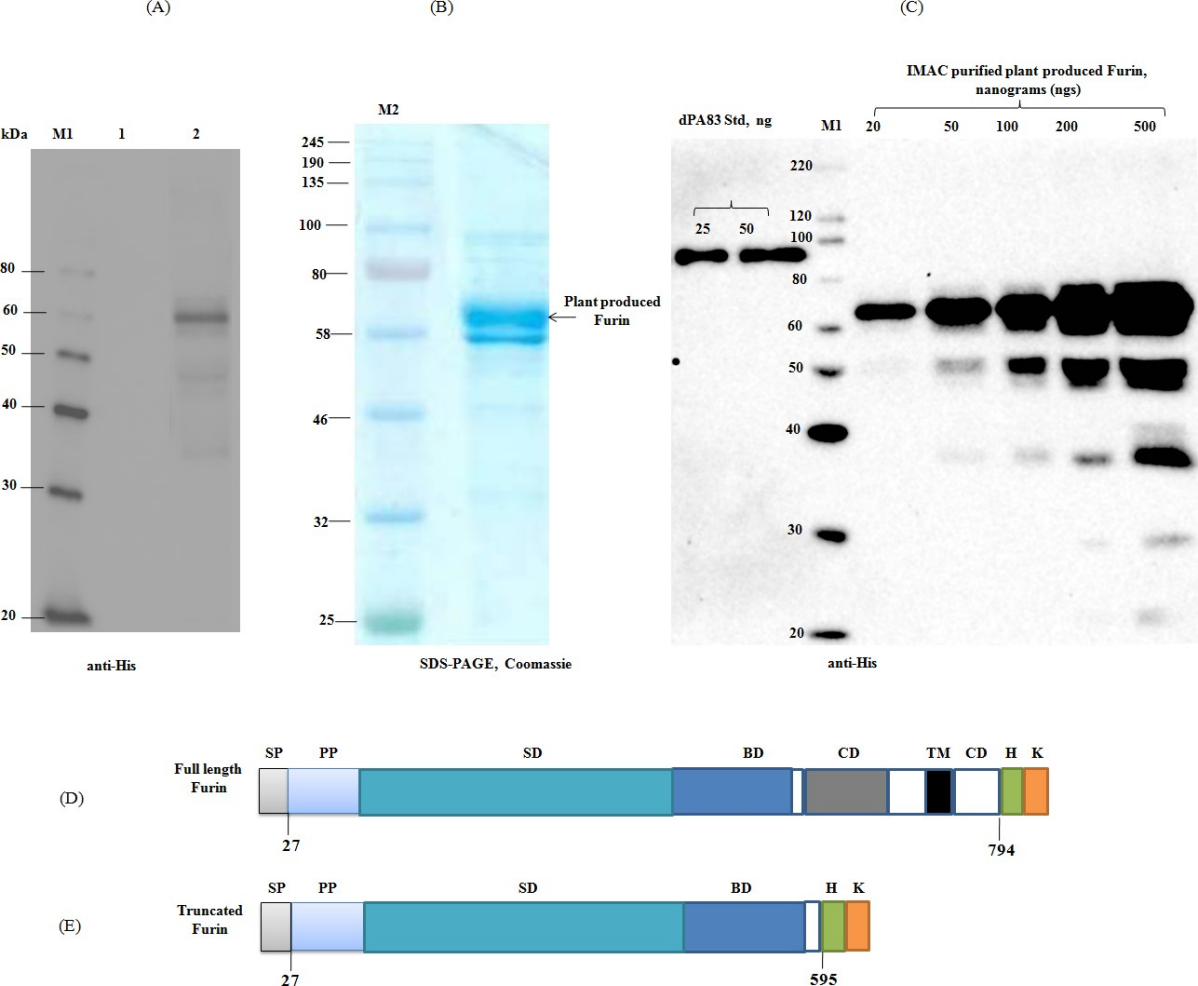
SDS-PAGE and Western blot analysis of human furin, produced *in N*. *benthamiana plants*. (**A):** Western blot analysis of human furin, produced in *N*. *benthamiana plants*. *N*. *benthamiana* leaf samples were harvested at 6 dpi. Samples for western blot analysis were prepared as described in Materials and Methods. Proteins on the blot were probed with a purified mouse anti-His tag antibody. 1- crude extract from non-infiltrated *N*. *benthamiana*; 2- crude extract from *N*. *benthamiana* plant infiltrated with pEAQ-Furin (truncated)-His-KDEL construct. M: MagicMark XP Western Protein Standard. (**B):** SDS-PAGE analysis of Ni-NTA column purified plant produced recombinant human furin. 5 μg Ni-NTA column purified protein was loaded into well. (**C**): Western blot analysis of different dilutions of Ni-NTA column purified, plant produced recombinant human furin, along with protein standards. Partially purified, plant produced furin was diluted 2.5, 5, 10 and 25-fold and different amount of plant produced furin, as indicated, was run on SDS-PAGE, followed by western blot. Plant produced His tagged furin protein band was detected using a purified mouse anti-His tag antibody. The concentration of furin in Ni-NTA column purified samples was quantified using the gene tools software, Syngene Bioimaging. Plant produced Endo H deglycosylated, purified PA83 protein (dPA83) was used as a protein standard. M1: color prestained protein standard (NEB); M2: MagicMark XP Western Protein Standard (ThermoFisher Scientific). (**D, E**): schematic representation of the full length (D) and truncated furin (E) structures. SP- Signal peptide; PP- Propeptide; SD- Subtilisin-like catalytic domain; BD- Homo B domain; CD- Cysteine rich domain; TM- Transmembrane domain; CD- Cytoplasmic domain.

As described in Materials and Methods, the human furin gene was engineered for expression in *N*. *benthamiana* plants using *N*. *benthamiana* codons, synthesized de-novo at Biomatik Corporation. In this study, the furin gene was expressed as a full length or truncated form in *N*. *benthamiana*. First, we tried to co-express the full length human furin with PA83 protein in *N*. *benthamiana*. However, when full length furin was co-expressed with the PA83 protein in *N*. *benthamina* plants there was little or no cleavage of PA83 (data not shown). In addition, very little or no specific furin protein band was detected in western blot when crude extract prepared from plants infiltrated with furin construct was probed with the specific antibody (data not shown). We therefore engineered a truncated form of human furin to produce a functionally active and highly soluble recombinant furin in *N*. *benthamiana*, using plant based transient expression system. Fig 1A demonstrates the confirmation of the production of human furin in *N*. *benthamiana* by western blot analysis. Fig 1A, 1B and 1C demonstrate that, plant produced, truncated human furin migrates as a ~60 kDa protein as expected. In order to examine the functional cleavage activity of human furin *in vitro*, recombinant human furin was purified using Ni-NTA column as described in Materials and Methods. SDS-PAGE and western blot analyses of Ni-NTA purified, plant produced recombinant furin is demonstrated in Fig 1B and 1C, respectively. As can be seen from Fig 1B and 1C, two bands of plant produced furin were observed in the gel, perhaps due to the presence of multiple glycosylated forms (non-glycosylated, high mannose N-glycosylation, differences in O-glycosylation [32], or the presence of furin with unoccupied N-glycan sites, where one or two glycosylation sites (out of 3) are not occupied by glycan). Protein bands that were observed on the blot at ~50 kDa, ~37 kDa and smaller (Fig 1C), are probably due to the proteolytic degradation of plant produced furin.

### Assessment of the in vitro cleavage activity of plant produced furin using an APRIL substrate protein

**Fig 2:**
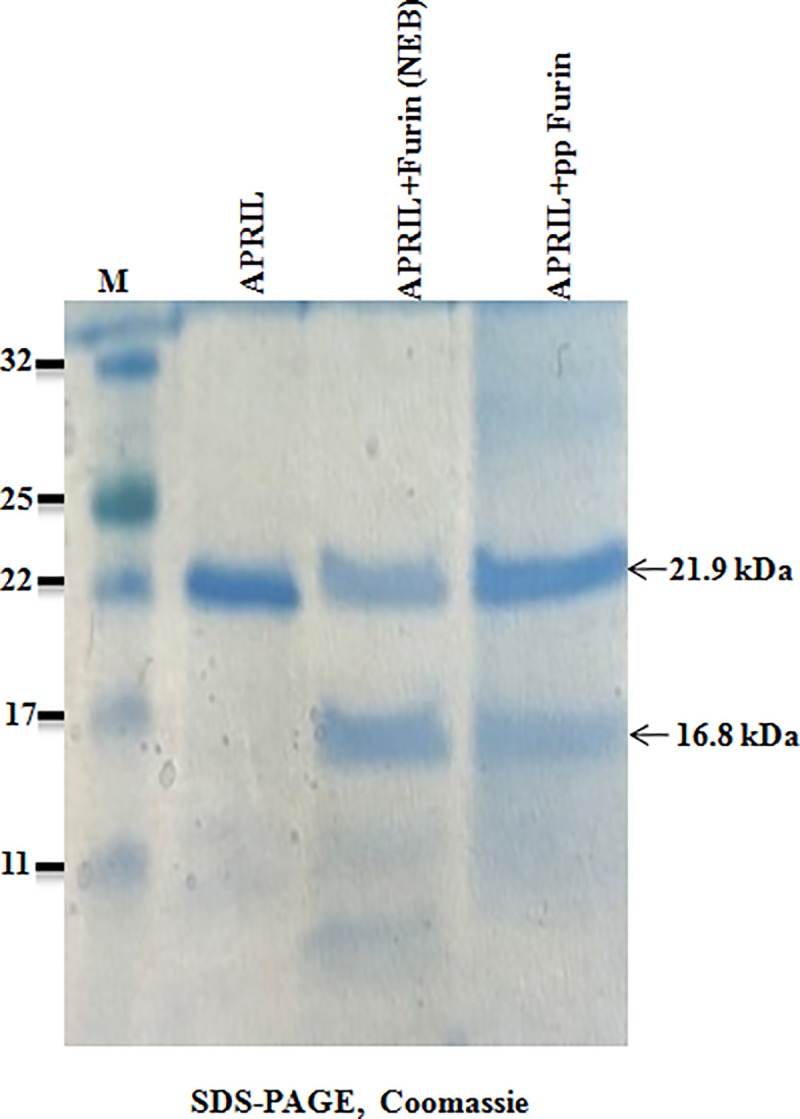
SDS-PAGE CBB analysis of recombinant APRIL protein, cleaved by plant produced or commercial Furin. 5 μg recombinant APRIL protein was incubated with 25 ng of plant produced human furin or 25 ng of commercial human furin (NEB) at 25°C, for 2 h. 2.5 μg APRIL protein from each sample was loaded in each well. M: color prestained protein standard (NEB).

The commercially available APRIL protein was used to evaluate *in vitro* cleavage activity of plant produced recombinant furin. APRIL is a proliferation-inducing ligand and a member of the tumor necrosis factor superfamily of proteins, which regulate immune responses and induces apoptosis [33]. The recombinant murine APRIL is a 21.9 kDa protein. The post-translational clevage by furin, results in the release of a C-terminal 16.8 kDa polypeptide fragment. To test the cleavage activity of plant produced furin, recombinant APRIL protein was incubated with plant produced human furin or commercial human furin (NEB) as described in Materials and Methods. Recombinant APRIL protein was cleaved by both plant produced and commercial furin resulting in the 16.8 kDa fragment (Fig 2). These results confirm that plant produced human furin is enzymatically active *in vitro* and specifically cleaves the APRIL protein.

### Assessment of in vitro cleavage activity of plant produced furin using the substrate protein, PA83

**Fig 3:**
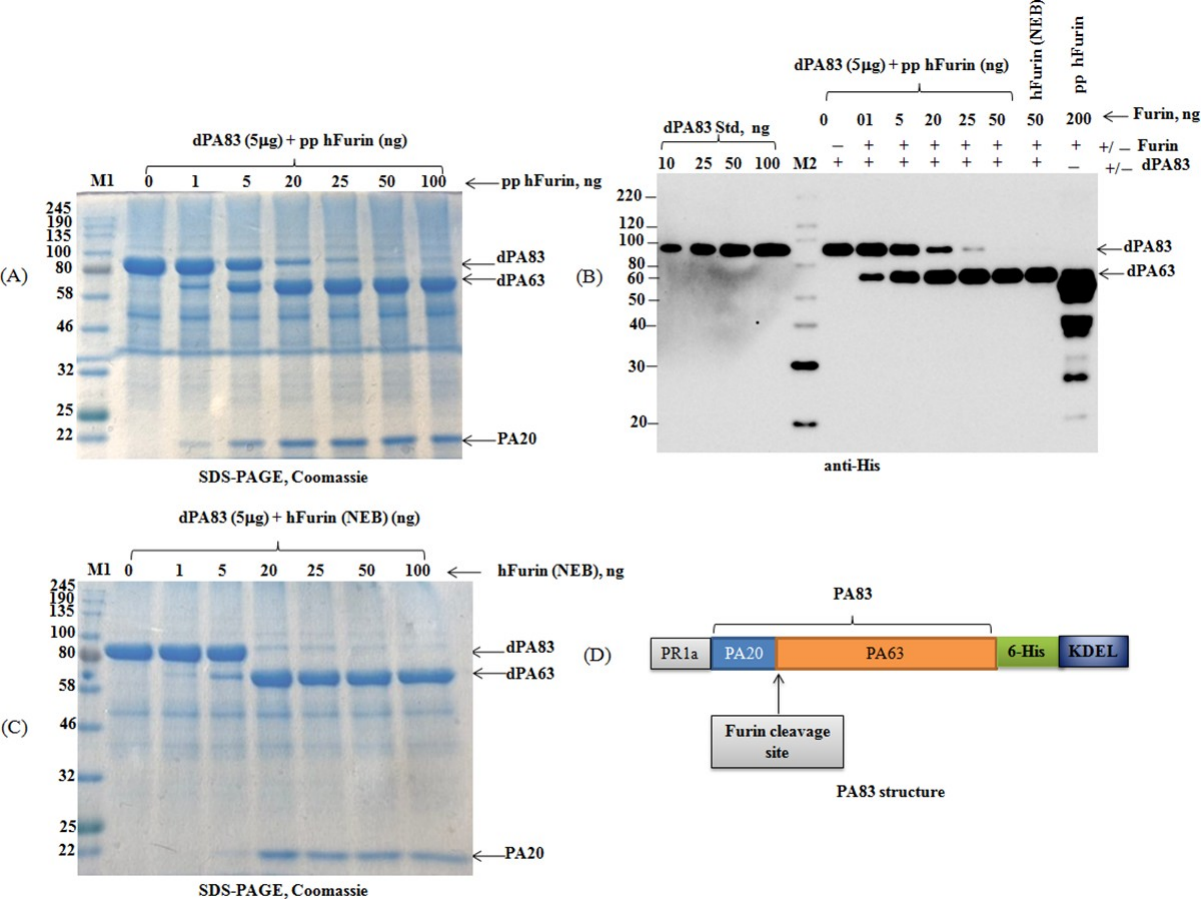
**SDS-PAGE CBB (A, C) and Western blot (B) analysis of dPA83, cleaved with plant produced or commercial human Furin. (A)**: 5 μg dPA83 (deglycosylated PA83) samples were treated with different concentrations (0, 1, 5, 20, 25, 50 and 100 ng) of plant produced human furin and then 4.5 μg samples were run on SDS-PAGE. dPA83: deglycosylated PA83; pp hFurin: plant produced, Ni-NTA column purified furin. M1: color prestained protein standard (NEB). (**B)**: 5 μg of deglycosylated PA83 samples were treated with different concentrations of plant produced human furin or 50 ng commercial (NEB) human furin as indicated, and then 100 ng PA samples were loaded into the gel. dPA83: deglycosylated PA83; pp hFurin: plant produced, Ni-NTA column purified furin. M2: MagicMark XP Western Protein Standard. **(C)**: 5 μg dPA83 (deglycosylated PA83) samples were treated with different concentrations (0, 1, 5, 20, 25, 50 and 100 ng) of commercial human furin (NEB) as indicated, and then 4.5 μg samples were run on SDS-PAGE. (**D)**: Schematic representation of PA83 protein structure. PA63 and PA20 (a 20-kDa amino-terminal fragment) are cleavage products of PA83 by furin. M1: color prestained protein standard (NEB).

To assess *in vitro* cleavage activity of plant produced furin, plant produced recombinant PA83 was used as a furin substrate. Protective antigen (PA83) is an 83 kDa protein that is secreted by the Gram-positive bacterium *Bacillus anthracis*, which binds the anthrax toxin receptor. After binding to the receptor, the PA83 antigen is cleaved by furin, the products (PA63 and PA20) localize to the cell surface, and are released into the extracellular environment [25]. PA83 protein (deglycosylated) was selected as suitable furin cleavage substrate as it generates two protein fragments with distinctly different molecular masses of 63 kDa (PA63) and 20 kDa (PA20) (see Fig 3D below), that could be easily visualized using SDS-PAGE. To test the cleavage activity of plant produced furin, a plant produced PA83 protein was incubated with different concentrations of purified furin as described in Materials and Methods. Protein samples were analyzed by SDS-PAGE (Fig 3A) and western blot analysis (Fig 3B). As shown in Fig 3A and 3B increasing the concentration of plant produced furin, the generation of PA63 and PA20 fragments are increased. Fig 3A and 3B show that 5 μg plant produced PA83 protein is about 85% cleaved by 25 ng plant produced furin resulting in the formation of the respective PA63 and PA20 fragments. As shown in Fig 3C, SDS-PAGE demonstrates that plant produced PA83 protein, is almost fully cleaved by a 20 ng of commercial furin (NEB). Based on these data, plant produced truncated furin is enzymatically active displaying about 75% relative activity compared to commercial human furin *in vitro*.

### Assessment the *in vivo* cleavage activity of plant produced furin by co-expression with FIX protein

**Fig 4:**
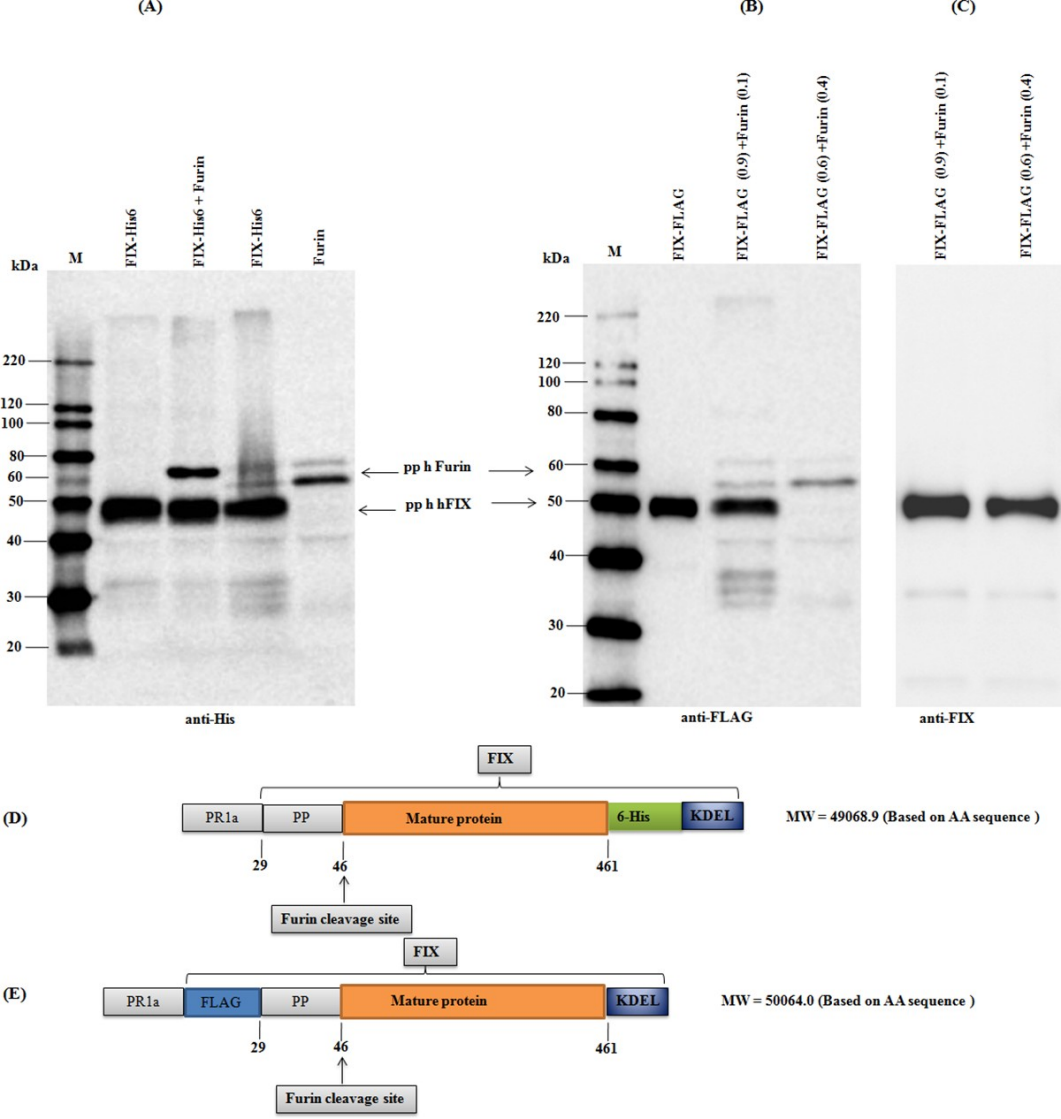
Western blot analysis of FIX, *in vivo* co-expressed with plant produced human Furin. (**A**): Samples were loaded as indicated. Proteins on the blot were probed with a purified mouse anti-His tag antibody. (**B)**: Samples were loaded as indicated. Proteins on the blot were probed with anti-FLAG antibody. (**C)**: Proteins on the blot were probed with anti-FIX antibody. (**D)**: Schematic representation of the FIX-His-KDEL construct structure. (**E)**: Schematic representation of the FLAG-FIX-KDEL construct structure. M: MagicMark XP Western Protein Standard.

FIX is expressed as a precursor polypeptide that requires posttranslational processing to yield a mature protein [34]. This precursor polypeptide of FIX undergoes several post translational modifications (PTMs), including the removal of the signal peptide (aa 1–28); carboxylation of the first 12 glutamic acid residues downstream from the18-amino acid propeptide sequence (aa 29–46) in the region rich in glutamic acid (aa 47–92, called the γ-carboxyglutamic acid or “Gla” domain) at the N terminus. Proper γ-carboxylation of the Gla domain is required for binding to calcium and phospholipids that is critical for proper protease activity during coagulation [34]. *In vivo*, vitamin K-dependent gamma-carboxylase binds to the 18-amino acid propeptide of FIX, which is then cleaved and is required for optimal binding of the Gla domain to Ca^++^ and phospholipids. When overexpressed in CHO cells, furin facilitates propeptide cleavage of FIX even when the recombinant protein is over-expressed at very high levels [35–37]. Therefore, expression of an active propeptide processing enzyme of furin in plants is critical for the production of functionally active FIX in plants. We performed *in vivo* co-expression of His6-tagged human FIX with human furin in *N*. *benthamiana* plants for possible cleavage of FIX by plant produced furin. The results are demonstrated in Fig 4. As can be seen from Fig 4A, since furin cleavage removes a fragment with 18-amino acid residues (Fig 4D), no visible shift in the molecular mass was observed. Therefore, we engineered a new FIX construct, with FLAG epitope at the N-terminal (Fig 4E). When FIX construct was co infiltrated with furin as a mixture at a ratio of OD_600_ (optical density, OD600) 0.9 (FIX) and 0.1 (furin), the FIX protein was detected with anti-FLAG antibody (Fig 4A). When FIX and furin were co infiltrated at a ratio of OD_600_ 0:6 (FIX) and 0.4 (furin) no FIX band could be detected with anti-FLAG antibody (Fig 4B). However, when the blot was probed with anti-FIX antibody, FIX bands were observed in both samples (Fig 4C). This confirms the loss of the FLAG epitope of the FIX protein was a result of furin cleavage. These data demonstrate that plant produced human furin is functionally active *in vivo* and specifically cleaved the plant produced FIX in plant cells.

### Assessment of the *in vivo* cleavage activity of plant produced furin by co-expression with PA83

**Fig 5:**
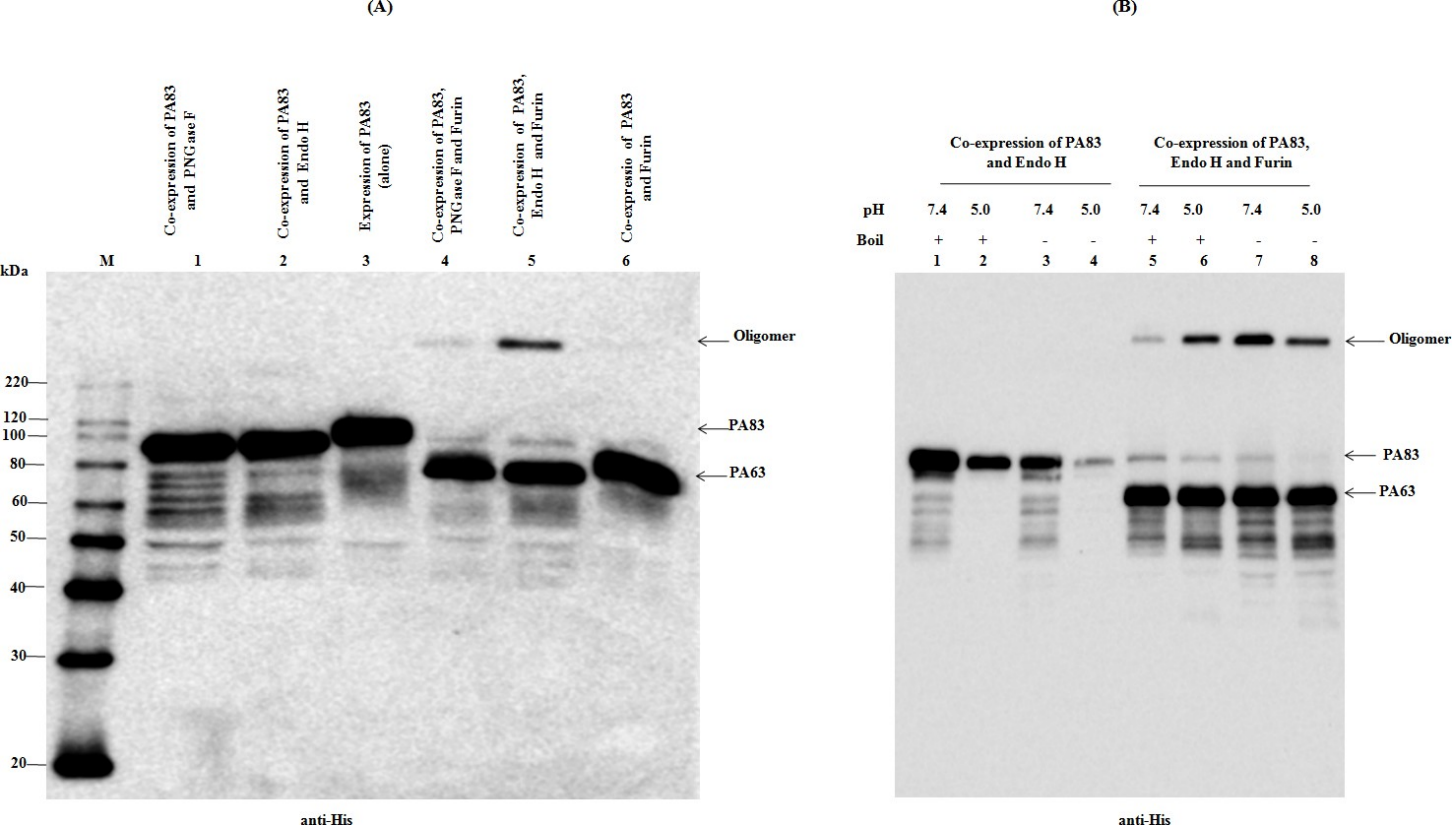
Western blot analysis of co-expression of PA83 with Furin, with or without deglycosylating enzymes Endo H and PNGase F in *N*. *benthamiana* plant. (**A**): Lanes: 1- Co-expression of PA83 with PNGase F for the production of PNGase F deglycosylated PA83 protein; 2- Co-expression of PA83 with Endo H for the production of Endo H deglycosylated PA83 protein; 3-Expression of PA83 **(**alone) for the production of glycosylated PA83 protein; 4- Co-expression of PA83 with furin and PNGase F for the production of furin cleaved and PNGase F deglycosylated PA83 protein; 4- Co-expression of PA83 with furin and Endo H for the production of furin cleaved and Endo H deglycosylated PA83 protein; 5- Co-expression of PA83 with furin for the production of furin cleaved and glycosylated PA83 protein. (**B**): WB analysis of *N*. *benthamiana* plant, infiltrated with PA83 and Endo H or infiltrated with PA83, Endo H and Furin constructs. 6-7-week-old *N*. *benthamiana* plant leaves, were infiltrated with the above constructs, were harvested and samples were processed for SDS-PAGE and western blot, as described in Materials and Methods. Boiled and un-boiled (raw) samples were diluted 5-fold and 10 μl from each sample was run on SDS-PAGE prior to western blotting. Proteins were probed with the purified anti-His tag antibody. The image was taken using a highly sensitive GeneGnome XRQ Chemiluminescence imaging system. An arrow indicates the protein bands corresponding to PA83 and PA63 and the formation of PA63 oligomers. M: MagicMark XP Western Protein Standard.

As mentioned above, *B*. *anthracis* PA is an 83-kDa protein (PA83) and after binding to receptors, PA83 is cleaved by cell surface furin and the 63 kDa (PA63) and 20 kDa (PA20) polypeptides are released [25, 38] and cell-bound PA63 then rapidly heptamerizes to form a pre-pore [39]. The *in vivo* cleavage activity of the plant produced furin was evaluated by co-expression with PA83 of *B*. *anthracis*. We also co-expressed PA83 with furin and deglycosylation enzymes Endo H or PNGase F to produce furin cleaved and deglycosylated PA83 proteins *in vivo*. The *in vivo* cleavage activity of the plant produced furin was evaluated by co-expression with PA83 and deglycosylation enzymes as follows: i) two genes, a human furin gene was co-expressed with PA83 to generate furin cleaved PA83 protein *in vivo*; ii) three genes, plant produced furin, Endo H and PA83 genes were co-expressed *in vivo* to generate furin cleaved and Endo H deglycosylated PA83 protein; iii) three genes, plant produced furin, PNGase F and PA83 genes were co-expressed to generate furin cleaved and PNGase F deglycosylated PA83. It should be noted that both PNGase F and Endo H are deglycosylation enzymes, secreted by the Gram-negative bacterium *Flavobacterium meningosepticum* and Gram-positive bacterium *Streptomyces plicatus*, respectively [40–42]. PNGase F cleaves asparagine-linked high-mannose, hybrid and complex oligosaccharides from glycoproteins, except oligosaccharides containing a (1–3)-linked core fucose that are commonly found in plant glycoproteins. Endo H is an enzyme that cleaves oligomannose-type and hybrid glycans, however, it does not cleave the complex N-linked glycans [43]. PNGase F deglycosylation removes the oligosaccharide intact, but results in an amino acid change due to the deamidation of asparagine to aspartate in the N-X-S/T site. Endo H catalyzes the cleavage between two N-Acetyl-D-glucosamine (GlcNAc) residues of the chitobiose core of N-linked glycans, and leaves single GlcNAc moiety, with no concomitant deamidation of asparagine [42]. Functionally active recombinant PNGase F and Endo H enzymes were recently produced in *N*. *benthamiana* plants. The *in vivo* deglycosylation by PNGase F and Endo H were successfully applied to the production of pharmaceutically important recombinant non-N-glycosylated proteins in plants [4, 44–46]. Notably, it was shown that the deglycosylation efficiency of plant produced Endo H was similar to those of commercial Endo H (NEB) *in vitro*, however, was greater than that of plant produced recombinant PNGase F *in vivo* [4]. Western blot analysis of PA83 proteins, co-expressed with modifying enzymes (furin, Endo H and PNGase F) are presented in Fig 5. In Fig 5A, if we compare PA83 protein, expressed alone (Fig 5A, lane 3) with PA83, co-expressed with furin (Fig 5A, lane 6), a shift of about 20 kDa resulting from a size reduction is observed. Similarly, if compare PA83, co-expressed with PNGase F (Fig 5A, lane 1), with PA83, co-expressed with PNGase F and furin (Fig 5A, lane 4) the same shift is observed. This 20 kDa size reduction is also evident if we compare PA83, co-expressed with Endo H (Fig 5A, lane 2) with PA83, co-expressed with Endo H and furin (Fig 5A, lanes 5). Finally, if we compare PA83 protein, expressed alone (Fig 5A, lane 3) with PA83 co-expressed with Endo H (Fig 5A, lane 2) or PNGase F (Fig 5A, lane 1) a gel shift is also observed due to deglycosylation of PA83 molecule (Fig 5A, lane 1 and 2), which is consistent with our previously published work [4]. All of the above results demonstrate that the PA83 protein undergoes two modifications *in vivo*: i) it is successfully cleaved by plant produced furin and ii) successfully deglycosylated by plant produced bacterial Endo H or PNGase F. As shown in Fig 5, a high molecular mass band (Fig 5A, as indicated and lane 5) was observed in plant-produced Endo H deglycosylated and furin cleaved PA83, suggesting heptamerization of furin cleaved and Endo H deglycosylated PA63 *in vivo*. As can be seen in Fig 5A, a high molecular band was also observed with PNGase F deglycosylated PA83 (Fig 5, lane 4), but not with glycosylated PA83 (Fig 5A, lane 6), suggesting that formation of the heptamerized form of glycosylated PA63 is probably blocked by plant N-linked glycans. This could explain why plant produced glycosylated PA83 has no biological activity and could not form lethal toxin (LeTx) *in vitro* [32, 44]. It should be noted that PA83 protein is not glycosylated in its native host, but contains six potential N-linked glycosylation sites that is aberrantly glycosylated when expressed in plants, and aberrantly glycosylated PA83 has no biological activity, and cannot form lethal toxin [44]. It was shown that acidic pH promotes oligomerization, which is SDS-resistant [45]. We, further tested the effect of pH on oligomer formation in boiled and un-boiled samples of PA63 protein, produced in *N*. *benthamiana* plants by co-expressing of PA83, Endo H and furin constructs. Boiled and un-boiled samples were analyzed by western blotting. In Fig 5B, high molecular mass proteins (oligomers) were not detected in the samples, prepared from *N*. *benthamiana* plants, infiltrated with PA83 and Endo H constructs (Fig 5B, lanes 1–4), however, they were detected in the samples, prepared from *N*. *benthamiana* plants, infiltrated with PA83, Endo H and furin constructs (Fig 5B, lanes 5–8). These findings suggesting that formation of high molecular mass oligomers in these samples is likely due to the furin processing of PA83 and production of PA63. The formation of PA63 oligomer is higher at pH 5.0 in both boiled and un-boiled samples (Fig 5B, lanes 6,8), and also is higher at pH 7.4 in un-boiled samples, but significantly lower at pH 7.4 in boiled sample, suggesting that the dissociation of oligomer occurs at pH 7.4 in conjunction with boiling in SDS sample buffer. Overall, these results are consistent with previously published work [45], suggesting the heptamerization of furin cleaved, Endo H deglycosylated PA63 occurs *in vivo* in *N*. *benthamiana* plants.

### Co-expression of furin with Endo H and PNGase F *in vivo*. Assessment of the cleavage activity of plant produced, deglycosylated furin *in vitro*

**Fig 6:**
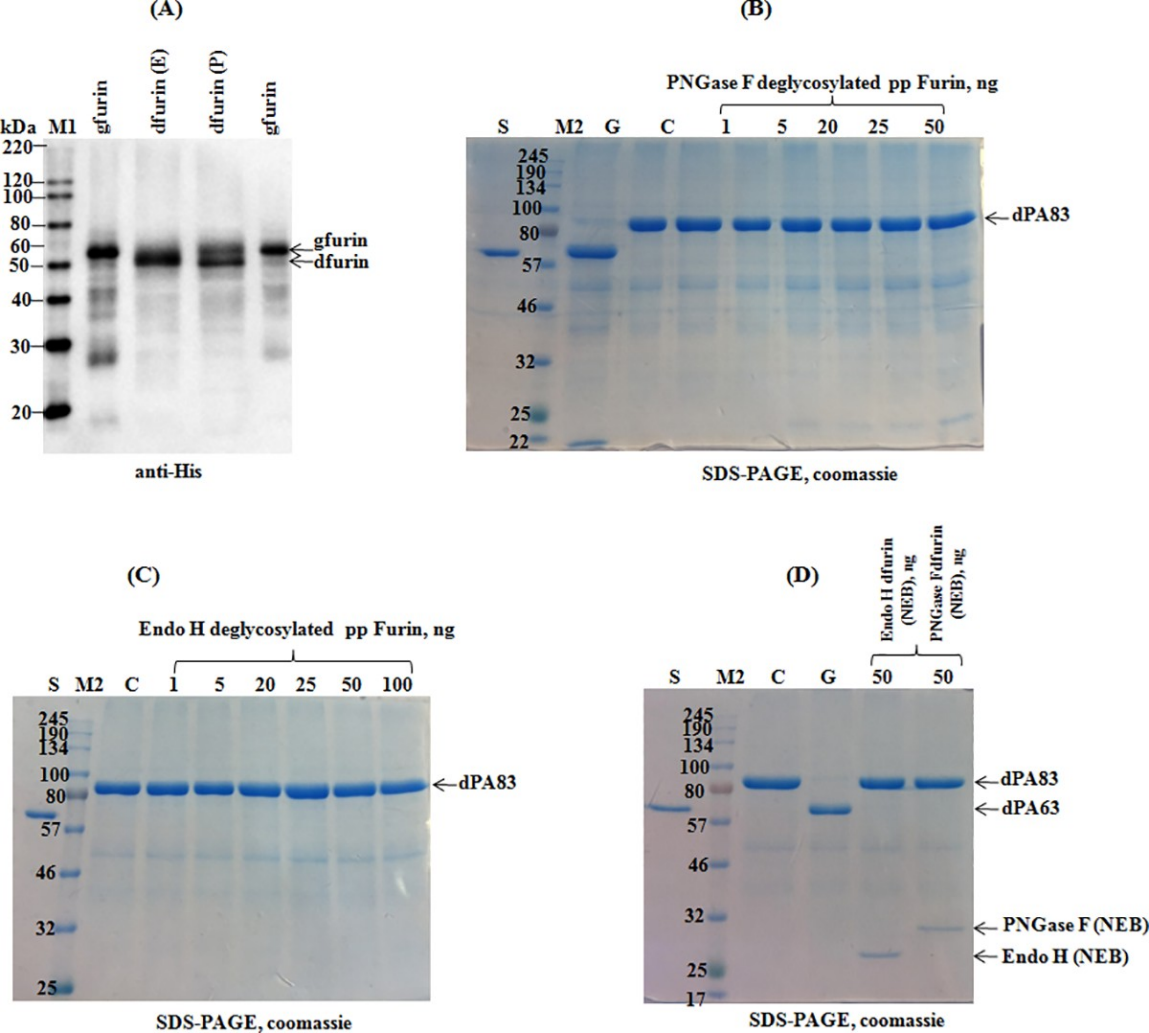
SDS-PAGE analysis of co-expression of human Furin with bacterial Endo H or PNGase F in *N*. *benthamiana* plant and evaluation of their cleavage activity. (**A**): Western blot analysis of Ni-NTA purified plant produced furin variants, i.e. glycosylated Endo H or PNGase F *in vivo* deglycosylated, as indicated. gfurin: plant produced furin, expressed alone (glycosylated); dfurin (E): plant produced furin co-expressed with Endo H; dfurin (P): plant produced furin co-expressed with PNGase F. Furin protein bands were detected using the purified anti-His tag antibody. M1: MagicMark XP Western Protein Standard. (**B**) SDS-PAGE CBB: 5.0 μg plant produced dPA83 was incubated with different amount (1.0, 5.0, 25, 50, 100 ng) of furin, which was co-expressed with PNGase F and then 2.5 μg PA83 protein from each sample was loaded into each well. G- positive control, 5.0 μg plant produced dPA83 was incubated with 50 ng of plant produced furin (glycosylated) and then 2.5μg dPA83 was loaded into a well and run on a SDS-PAGE. C-negative control, plant produced dPA83 protein, not incubated with furin. M2-color prestained protein standard (NEB); S- BSA standard. (**C**): 5.0 μg plant produced dPA83was incubated with different amount (1.0, 5.0, 25, 50, 100 ng) of furin co-expressed with Endo H and then 2.5 μg PA83 protein from each sample was loaded into each well. C-negative control, plant produced dPA83 protein, not incubated with furin. M2-color prestained protein standard (NEB); S- BSA standard. (D): 5.0 μg plant produced PA83 (deglycosylated) was incubated with commercial human furin, which was previously in vitro deglycosylated with commercial Endo H (lane 2) or PNGase F (Lane 3). Lane 1, positive control, 5.0 μg plant produced dPA83 was incubated with 50 ng commercial human furin (NEB) and 2.5μg PA83 was loaded into a well. C-negative control, plant produced dPA83 protein, not incubated with commercial furin. M2-color prestained protein standard (NEB); S- BSA standard.

Human furin protein has three potential N-glycosylation sites, Asn (387), Asn (440) and Asn (553). The human furin gene was co-expressed with both Endo H or PNGase F to produce deglycosylated forms of the enzyme. Co-expression of furin with Endo H or PNGase F reduced the molecular mass of furin (Fig 6A, as indicated), compared with non-deglycosylated forms, suggesting the *in vivo* deglycosylation of plant produced furin. Fig 6A shows that, a complete deglycosylation was observed when furin co-expressed with Endo H, but was not completely deglycosylated when co-expressed with PNGase F. These results are consistent with our recent findings that deglycosylation efficiency of plant produced Endo H is greater than that of plant produced PNGase F [4].

To test the effect of *in vitro* deglycosylation on furin cleavage activity, furin proteins that were co-expressed with Endo H and PNGase F were purified from plant tissue using the Ni-NTA column as described in Materials and Methods above. Fig 6 demonstrates a SDS-PAGE analysis of plant produced dPA83 incubated with plant produced or commercial furin (NEB). As can be seen in Fig 6, plant produced human furin treated with PNGase F (Fig 6B) and Endo H (Fig 6C) does not exhibit cleavage activity. Similarly, when commercial human furin (NEB) was treated with commercial Endo H or PNGase F (Fig 6D), no cleavage was observed for the substrate PA83. These results demonstrate that N-glycosylation is necessary for the proper folding and catalytic activity of plant produced or commercial human furin (NEB). It should be noted that the effect of deglycosylation on the enzymatic activity of human furin has not been reported previously. However, previous studies have shown that site-directed mutagenesis of two N-glycosylation sites (Asn387 and Asn440), negated furin activation [46]. In contrary to *in vitro* deglycosylated furin (plant produced or commercial furin), plant produced furin was fully active *in vivo* and successfully cleaved PA83 protein, when PA83 was co-expressed with Endo H or PNGase F and furin. This resulted in a ~20 kDa size reduction of PA83 and the formation of PA63 protein (Fig 5). A possible explanation for this discrepancy may reflect potential differences in protein folding *in vitro* and *in vivo*, as molecular chaperones assist protein folding. Notably, our previous studies [4, 44] showed that even though PA83 proteins treated with PNGase F either *in vitro* or *in vivo* have identical amino acid sequences, the lethal toxin neutralizing activity and immunogenicity of plant produced *in vivo* deglycosylated PA83 was greater than that of *in vitro* deglycosylated PA83 [44]. In addition, our recent studies demonstrated that the plant produced form of PA83 deglycosylated *in vivo* by Endo H appeared to be more stable at elevated temperatures compared to form treated with Endo H *in vitro*, suggesting potential differences in protein folding of *in vivo* and *in vitro* deglycosylated forms [4].

## Discussion

Recently a series of strategies and approaches have been utilized to successfully develop a system for the efficient recombinant production and subsequent PTM of proteins in plants, which are important for proper folding, functionality and stability [4, 44, 47, 48]. In this study, we engineered the human furin gene for expression in *N*. *benthamiana* plants resulting in the production of a highly soluble, functionally active enzyme. We show that plant produced furin has about 75% relative activity of commercial human furin *in vitro*. The major goal of this study was to achieve furin processing in plants and apply the technology to the PTM of proteins, in which furin cleavage is necessary for maturation and activation. Moreover, we demonstrate that plant produced truncated form of human furin is active *in vivo* and specifically cleaved target proteins, FIX and PA83. As described above, furin modifies proteins of vitamin K-dependent coagulation Factors (Factors VII, IX and protein C). Defects in FIX synthesis result in hemophilia B (Christmas disease), an X-linked disorder. Currently, patients with hemophilia B are mainly treated with FIX, obtained from concentrates made from human blood and recombinant FIX produced in CHO cells. However, such preparations of FIX are extremely expensive and difficult to obtain, especially in developing countries. To date, all attempts at producing recombinant Factor IX using different expression systems have been hampered by limitations in post-translation processing, safety concerns, and high cost. There remains an urgent need for a safe and affordable therapeutic treatment for hemophilia B. Thus, the findings in this study may open the door for the production of affordable, safe (pathogen free), functionally active human clotting factors, such as FIX, Factor VII and protein C in plants using transient expression technology. In this study, we also demonstrate that both enzymatic deglycosylation and proteolytic processing of PA83 protein were achieved *in vivo* by introducing the respective enzymatic repertoire into a eukaryotic system. The PA83 protein does not carry N-linked glycans in the native hosts, but contains potential N-linked glycosylation sites, which are aberrantly glycosylated during expression in plants [4, 44, 47, 48]. Glycosylated PA83 has no biological activity, and therefore, cannot form a lethal toxin [32, 44]. It has low immunogenicity compared to the deglycosylated form [4, 32, 44], and is also highly unstable especially at elevated temperatures [4]. Thus, deglycosylation of PA83 or PA63 is important for functional activity. Plant produced, PNGase F [44, 47] or Endo H deglycosylated forms of PA83 [4] were more stable compared to the glycosylated counterpart and had a superior immunogenicity. However, further improvement in the potency, immunogenicity and stability of the anthrax vaccine is still needed. *In vivo* processing of PA83 the protein and production of deglycosylated PA63 protein in plants could potentially be used to develop a new vaccine candidate against anthrax, based on heptamerized PA63, which can be produced *in vitro* (without the need of costly commercial cleavage enzymes, i.e. furin or trypsin) or *in vivo*. This technology has potential applications in molecular farming and can be used to produce subunit vaccines, therapeutic proteins, and antibodies in eukaryotic system. Recombinant human furin has not been previously produced in plants, therefore, the technology developed in this study supports the utility of plants as an efficient expression system for the production of active, endotoxin-free recombinant human furin.
